# Food sovereignty: an alternative paradigm for poverty reduction and biodiversity conservation in Latin America

**DOI:** 10.12688/f1000research.2-235.v1

**Published:** 2013-11-06

**Authors:** M Jahi Chappell, Hannah Wittman, Christopher M Bacon, Bruce G Ferguson, Luis García Barrios, Raúl García Barrios, Daniel Jaffee, Jefferson Lima, V Ernesto Méndez, Helda Morales, Lorena Soto-Pinto, John Vandermeer, Ivette Perfecto

**Affiliations:** 1 Institute for Agriculture and Trade Policy, Minneapolis, MN, 55404, USA; 2 School of the Environment and The Center for Social and Environmental Justice, Washington State University Vancouver, Vancouver, WA, 14204, USA; 3 Faculty of Land and Food Systems and Institute for Resources, Environment and Sustainability, University of British Columbia, Vancouver, V6T 1Z4, Canada; 4 Environmental Studies Institute, Santa Clara University, Santa Clara, CA, 95050-4901, USA; 5 Departmento de Agroecología, El Colegio de La Frontera Sur, Carretera Panamericana y Periférico Sur s/n, Chiapas, CP 29290, Mexico; 6 Centro Regional de Investigaciones Multidisciplinarias, Universidad Nacional Autónoma de México, Cuernavaca, CP 62210, Mexico; 7 Department of Sociology, Portland State University, Portland, OR, 97207-0751, USA; 8 Instituto de Pesquisas Ecológicas, Nazaré Paulista, Brazil; 9 Department of Plant and Soil Sciences, University of Vermont, Burlington, VT, 05405, USA; 10 Department of Ecology and Evolutionary Biology, University of Michigan, Ann Arbor, MI, 48109, USA; 11 School of Natural Resources and Environment, University of Michigan, Ann Arbor, MI, 48109, USA

## Abstract

Strong feedback between global biodiversity loss and persistent, extreme rural poverty are major challenges in the face of concurrent food, energy, and environmental crises. This paper examines the role of industrial agricultural intensification and market integration as exogenous socio-ecological drivers of biodiversity loss and poverty traps in Latin America. We then analyze the potential of a food sovereignty framework, based on protecting the viability of a diverse agroecological matrix while supporting rural livelihoods and global food production. We review several successful examples of this approach, including ecological land reform in Brazil, agroforestry, *milpa*, and the uses of wild varieties in smallholder systems in Mexico and Central America. We highlight emergent research directions that will be necessary to assess the potential of the food sovereignty model to promote both biodiversity conservation and poverty reduction.

## Introduction

At the 2012 Rio+20 meetings, political leaders acknowledged the mounting challenges to sustainable development, reiterating that many of the world’s poor depend on rapidly disappearing and fragile biodiverse ecosystems. In rural areas, poverty traps, defined as “self-reinforcing mechanisms that cause poverty, however measured, to persist” ^1, 2^ often result from linked ecological and socio-political systems that reach a dynamic equilibrium at a low level of human wellbeing. In relation to biodiversity, poverty traps raise the question of how to improve socio-economic wellbeing without further increasing the consumption of scarce, fragile, or overexploited resources. It has been argued, for example, that sustained improvements in well-being can be accomplished, but at a cost to biodiversity, or that in some situations conserving biodiversity would mean keeping a group of people at existing levels of poverty. Alternatively, there are also theoretical and empirical arguments that “win-win” situations can be found where fighting poverty and inequality may increase sustainability and biodiversity conservation ^3– 6^.

However, empirical and theoretical explorations of the relationships between poverty traps and biodiversity loss are largely underdeveloped. Little attention, for example, has been given to the exogenous socio-economic drivers of those poverty trap dynamics. Thus, Maru *et al.* ^2^ suggest rethinking current approaches, emphasizing the importance of “causes external to the system” in creating and maintaining poverty traps. For example, income improvements due to the rapid agricultural development of the 1960s and 70s did not reach the most impoverished sectors, exacerbating historical inequalities ^7^.

In this paper, we examine exogenous factors that contribute to poverty traps for smallholders in Latin America. We suggest a reconsideration of the role of neocolonial/neoliberal policies and agro-export models in addressing poverty: in Latin America, 52% of rural people still remain in poverty ^8^, with significant evidence linking both the maintenance of rural poverty and the environmental degradation at the agricultural frontier (e.g., biodiversity loss, erosion, deforestation) to agricultural intensification and the growing integration of agriculture into world markets ^9– 11^. We then re-examine the relationship between biodiversity and diverse small-scale farming systems, and present evidence that small-scale agroecological farms contribute to enhancing farmer's livelihoods and the conservation of biodiversity at local and landscape levels, as well as ecosystem services. We then assess the ability of an alternative food sovereignty framework to address the challenge of reducing poverty, improving food security and conserving biodiversity and other natural resources in Latin America. We suggest a reframing of the biodiversity loss and poverty trap dilemma and provide an approach for moving beyond the narrow land-sparing/land-sharing debate (e.g., Phalan *et al.* (2011) ^12^ and Tscharntke *et al.* (2012) ^13^) in the ongoing global search for how best to feed the world and reduce poverty, while protecting essential ecological services, including biodiversity.

### Contribution of exogenous factors to poverty and land degradation in Latin America

Development economics has long emphasized the strong interdependence between natural systems and human wellbeing, especially in rural areas. Conventional approaches have held that poor rural populations are involved in two vicious circles constituting a poverty trap: 1) the poor are unable or unwilling to regulate their numbers, which, on average, leads to surplus labor and further impoverishment; and 2) poverty leads to the depletion of soil organic matter and other forms of “mining the soil”, generating low productivity and deforestation and leaving those who depend on these resources for livelihoods in continued poverty ^3, 14, 15^. The policy prescriptions that follow are generally directed at stopping further increases in the population/labor surplus and consequently halting the depletion of natural resources. This broadly re-capitulates earlier Neo-Malthusian views, even though more recent work sometimes nods to more sophisticated analyses based in ideas of “upgrading human capital”: providing education and health programs, and direct welfare assistance ^16^. These “upgrades” are proposed as ways to break vicious circles between poverty, population, and environmental degradation, ignoring the fact that the “vicious circle” conceptualization itself is simplistic and problematic ^16, 17^.

Thus interventions formulated and implemented in this vein have often fallen far short of their desires to transform the rural poor into a sustainably productive sector. This is due to the extremely simplistic view of poverty dynamics represented by points (1) and (2) above. The rural poor in the capitalist world do not exist in a vacuum. Rather, they participate in complex institutional and economic arrangements involving market and non-market transactions at local and trans-local levels. Moreover, redistributive land reform (i.e., “actual net transfer of effective control” of land to poor peasants; ^18^) has important repercussions for rural livelihoods, hunger, poverty alleviation and biodiversity conservation in the region ^19, 20^. More technically stated, existing programs have neglected the combination of the lack of physical and human capital and distortions and failures in the product, labor and credit markets in which the rural poor operate, rendering them incapable of investing resources in ecosystem conservation and restoration. They may then become dynamically inefficient and uncompetitive producers, further restricting their capacity to acquire necessary new capital and overcome their economic disadvantages—in other words, caught in a poverty trap.

Further, as we will argue, rural poverty traps are also the result of exogenous factors, including the legacy of colonialism and the continuation of historical inequalities in agricultural and trade policies ^21, 22^. Various authors ^14, 17, 23– 28^ have documented a number of structural biases against poor rural households (as summarized by Taylor and García-Barrios (1999) ^16^:

“…unfavorable economic policies and public investment priorities (especially with the onset of the debt crisis in the 1980s); structural and institutional contexts that are unfavorable to rural development, including inegalitarian land tenure systems and institutional biases against smallholders in the definition of public goods and services and in their access to them; economic policies and technological biases that reduce employment creation in both the non-agricultural sector and in commercial agriculture; household-specific market failure, economic discrimination and adverse selection in the labor, product and credit markets; [government-abetted] monopolistic power in local formal and informal markets [that generates] compulsory transactions which, like usury, lead to the expropriation of their resources; [and] direct private and State coercive violence”.

Taylor and García-Barrios expand on this, arguing that the highly constrained, unfavorable situations facing the poor may compel what are (in these circumstances) economically rational survival strategies. However, these strategies and transactions easily move from being constrained *choices* to established, involuntary, and compulsory parts of the rural poor *habitus* (lifestyle, behavior, and worldview), ultimately maintaining or increasing the poor’s conditions of poverty and dependence. For example, peasant “brain drain” and “labor drain” may undermine local institutional arrangements by eroding social norms and capital. The structural conditions that emerge may generate local institutional insufficiency, systemically affecting the capacity of the poor to reorganize endogenously in the face of new challenges ^16, 29, 30^.

This broader set of explanations provides an understanding of poverty traps as multiple and embedded (fractal), and shows that traps resulting from actions by other human actors and socio-economic inequalities may be the norm, not the exception ^2, 31, 32^. As said by the World Bank, “even where poor people degrade the environment, this is often due to the poor being denied their rights to natural resources by wealthier elites and, in many cases, being pushed onto marginal lands more prone to degradation” ^33^, *Box 4*. This explanation, however, is still insufficient in two ways.

### Institutionalized disadvantages and the neoliberal paradigm

The first insufficiency is its lack of a clear assessment of the impacts of institutionalized competitive disadvantages on smallholder farmers, including, for example, international financial institution support for export-oriented commodity production and the liberalization of international agricultural trade ^34^. Such neoliberal agricultural development programs have purported to eliminate structural market failures and create favorable conditions for small farmers and their access to global markets ^35, 36^. Such policies, however, resulted in the liberalization and opening of Latin American economies, including the agricultural sector, and the dismantling of public services related to agriculture, such as credit for smallholders, technical support, etc. ^35^. But at the same time, beginning in the mid 1970s and extending through the 1980s and 1990s, the World Bank made it clear that their development programs envisioned two options for Latin American smallholders: 1) become commercial, export-oriented, farmers, or 2) disappear ^32, 37, 38^.

The results, however, were far from those intended: the smallholder sector in Latin America has not declined, as anticipated by development theorists, but has actually increased ^8, 36^. But though the peasant sector has remained, the challenges facing it have deepened: neoliberal agricultural policies have reinforced fractal poverty traps and deepened patterns of rural inequality; international and internal inequalities of market integration were propagated through multiple scales, with largely negative impacts on welfare in rural areas, including widespread rural displacement and cross-border migration ^19, 22, 39, 40^. Further, neoliberal policies resulted in the inequitable distribution of economic growth: despite an increase in GDP of 25% in real terms for the region, poverty and hunger barely improved, especially in the rural areas. In 1980, 60% of the rural population was poor and 33% suffered from hunger; in 2010 the percentages were 52% and 29%, respectively ^8, 19, 36^. Indeed, rural Latin America has the most unequal rural sector in the world, with Gini coefficients higher than 0.5 for most countries ^41^. Inequalities in land access, an important asset for rural households, are also the worst in the world, with an average (land-ownership-based) Gini coefficient of 0.78 for the region ^42^. Thus poor households that depend on agriculture as their primary source of income have been the most affected by neoliberal policies, with stagnation or deterioration in welfare over the past 20 years ^43, 44^.

In parallel to these dynamics, changes in agricultural technology and trade policies favoring export-oriented production have also been repeatedly tied to environmental degradation ^9, 45– 47^: the regional shift to export crops grown in monocultures has led to increased water and agrochemical uses, and has had detrimental impacts on biodiversity ^48– 52^; dramatic increases in the use of synthetic inputs (i.e., pesticides and fertilizers) have contributed to rapid, but currently tapering, yield increases worldwide ^53^; and agricultural industrialization has corresponded to increasing rates of deforestation, a massive movement of people from rural to urban environments, and an overall loss of biodiversity ^47, 54, 55^. Further, export-focused agriculture has often displaced land, research, and institutional support for crops grown for regional or national consumption, hurting small farmers’ livelihoods and food security more broadly ^46, 56– 58^. While it was hypothesized that the higher yields from agricultural intensification would allow less land to be used for agriculture and more land “saved” for biodiversity, evidence is also accumulating that higher yields rarely create this “land-sparing” effect ^59, 60^, and in fact may stimulate expansion of agricultural frontiers, including what has come to be known as the global “land grab”. Beyond this, higher yields do not assure increased access to food or decreases in poverty ^61– 64^. This approach is nevertheless manifest in the many programs designed to separate agriculture and nature as distinct land uses, a strategy with mixed results for conservation ^65^.

### Variations in the experiences of Latin American smallholders

The second source of insufficiency of the contemporary poverty trap discourse is that it does not explain the substantial variation of agricultural experiences in the region. Small-scale landholders still represent a large percentage of the agricultural landholdings in Latin America. In a study that included 15 Latin American countries, Chiriboga ^66^ estimated that their smallholder sectors were composed of 6 million semi-commercial family farms controlling 42% of the land, plus 11 million subsistence farms controlling 3% of the land. (Corporate farms were estimated to number around half a million and to control ~56% of all agricultural land). Because the smallholder sector is deeply embedded in local economies, their role in feeding the region and conserving the biota should not be underestimated. For example, the *World Development Report 2008: Agriculture for Development* marked a shift away from the focus on an export-oriented model and a recognition of the importance of small-scale agriculture in poverty reduction ^20^. The authors also recognized for the first time in almost 30 years the critical role of government in overcoming market failure ^67^. However, the report continues to call for deeper liberalization in agriculture, an approach that has repeatedly failed to address the deep poverty and inequality in Latin America ( ^19, 20, 22, 68^; for discussions of similar dynamics in other regions, see Moseley *et al.* (2010) ^69^ and Buckland (2006) ^70^). This connects to the insufficiency of contemporary discourse in that regional and local variations are rarely accounted for within the grand narratives of development discourse—the exact configurations of disadvantage, historical and exogenous drivers, institutional characteristics, interactions with local ecosystems, and therefore possible solutions are likely to vary, possibly immensely, from case to case, creating the need for approaches based in specific contexts of place and space ^31, 71– 73^. Expressed more technically, rural social dynamics, of which poverty traps are a result, are complex processes that may render multiple attractors and trajectories. The positive (self-reinforcing) but degrading feedback between poverty and land productivity suggests an alternative positive but upgrading feedback: biodiversity benefiting smallholders, and smallholders practicing diversified agroecology that benefits biodiversity. It has been argued that in contrast to heavily consolidated rural landscapes that have resulted from agricultural liberalization and export agriculture ^5, 22^, landscapes composed of mosaics of natural habitats and small-scale, diverse farms oriented toward local markets can also stimulate local economic development and reduce poverty in rural areas ^74– 78^. This possibility is the main object of analysis of this article, and to that we now turn.

## Relationships between biodiversity and smallholder agriculture

The evidence in support of an alternative and upgrading positive feedback loop between peasant production and biodiversity management is strong, although it also suffers from broad generalizations that have often emerged from small-scale (spatial and temporal) experimental studies ^79^. These caveats notwithstanding, the scientific consensus is that biodiversity is essential for agriculture and that agriculture, in turn, impacts biodiversity, both in positive and negative ways depending on the type of agriculture ^80^.

### Biodiversity's benefit to agriculture and rural livelihoods

Biodiversity is the basis of agriculture: it is the origin of all crops and domesticated animals from which humans derive their sustenance. Of the ~30,000 species of edible higher plants, it is estimated that ~7,000 have been cultivated. In addition to enabling the production of food across a wide spectrum of environmental conditions, crop diversity (especially fruits and vegetables) contributes to food security, a diversified diet and higher quality nutrition ^81– 83^. In addition to the provisioning services associated with crop and animal production, biodiversity can contribute to ecosystem services that benefit agriculture and society more generally. These include higher yield and overall production output through intercropping and agroforestry, regulation of pest and diseases, nutrient cycling though decomposition of organic matter, carbon sequestration, soil water retention, and pollination services. Although the literature on the relationship between biodiversity, ecosystem services, and agriculture is robust, it is not without controversy. For example, there is a strong debate about the relationship between biodiversity and productivity. While the advantages of intercropping are well-documented, in most cases the overyielding of intercrops as compared to monocultures is the result of the combination of a grass and a legume and not biodiversity *per se* ^84^. Likewise carbon sequestration or pollination services could, in theory, be maximized with the presence of the most efficient carbon sequestering plant or pollinator. However, for smallholder agriculture it is the diversity of crop and animal varieties, crops and animal species and wildlife that provide these ecosystem services under variable and changing environmental conditions ^13, 85^. Diverse agroecological systems also buffer the impacts of climate change ^86– 90^ and reduce the vulnerability of smallholders to price and market fluctuations ^91– 94^.

### Smallholder agroecological farms contribute to the conservation of biodiversity

Agriculture is recognized as one of the major drivers of biodiversity loss ^80^, mostly through habitat destruction, soil erosion, monocultures and the use of agrochemicals ^95^. But not all types of agriculture have the same effects on biodiversity. Diverse agroecological and organic systems have been shown to contribute to biodiversity conservation at the local and landscape level ^5, 96– 101^. At the local/farm level agroecological and organic systems can benefit biodiversity by eliminating the use of pesticides and other agrochemicals, increasing crop diversification and crop rotations, preserving hedges and other wild vegetation, and through soil conservation measures. Agrobiodiversity encompasses genetic resources, edible plants and crops, and livestock (planned biodiversity), as well as the associated organisms (associated biodiversity) that provide ecosystems services such as maintenance of soil fertility and prevention of pest attacks ^102^. Higher associated biodiversity is strongly correlated to planned biodiversity, meaning more diverse agricultural systems generally maintain greater levels of ecosystem services and landscape diversity ( ^103– 105^, but see Balmford *et al.* (2005) ^106^). In a meta-analysis that included 63 publications comparing organic and conventional farms, Bengtsson and colleagues ^98^ reported that, on average, organic farming increases species richness by 30% and organism abundance by 50% over conventional farming. Although the results were variable, and not all organisms responded in the same way, their meta-analysis provides evidence that organic farming generally supports higher levels of species richness, especially of plants, birds and predatory insects, than conventional agriculture ^98^ Other reviews and meta-analyses have arrived at the same conclusion ^95, 107– 113^. In a more recent synthesis, Kremen and Miles ^101^ suggest that diversified farming systems enhance ecosystem service provisioning including biodiversity conservation, fostering agro-ecosystem resilience and sustainability.

The benefits to biodiversity of certain agri-environmental schemes in Europe have been questioned ^114^, with many examples where intended or hoped-for biodiversity benefits have not materialized ^115^. However, it has been proposed that these schemes may not have delivered greater biodiversity benefits because they are typically designed for the farm- or field-scale and frequently ignore the surrounding landscape ^116^, and because they may not be using appropriately researched and designed wildlife-friendly methods ^100^. At a larger scale landscape heterogeneity is an important factor in maintaining biodiversity ^95, 103, 105, 116– 124^, and can be as or more important than the type of management at the farm level ^125^. The tendency in the conventional agriculture model, however, has been to reduce diversity not only at the farm level but also at the landscape level ^95^. Furthermore, entire landscapes are tending toward homogenization under current policies, which tend to promote larger farms characterized by large monocultural fields with fewer non-cultivated habitats: live fences, non-cultivated field margins, hedge rows, and scattered trees ^125– 128^, and thus exacerbate the negative effects of agricultural intensification on biodiversity ^129^.

At the individual farm scale, researchers are just beginning to examine the effect of farm size *per se* on biodiversity. A study of farms of various sizes and management types (organic and conventional) in Sweden reported that, although organic farms had higher diversity than conventional farms, the biggest differences were found between small organic and large conventional farms ^125^. The same study also found 56% more bird species in small versus large organic farms, suggesting that landscape level factors were playing an important role for bird diversity and that size matters. At least two other studies have reported that field size is an important factor affecting biodiversity ^130, 131^. Landscape configurational heterogeneity (i.e., pattern complexity; see ^116^) can also be important. For example, when Fahrig *et al.* ^116^ compared fine-grain and coarse-grain landscapes in France (that is, landscapes with smaller fields and shorter distance between hedgerows versus landscapes with larger fields but similar crop types) they found that carabid beetle species richness accumulated faster in the fine-grain landscapes. And as Fahrig *et al.* point out, similar results were reported for solitary wasps in Germany ^132^.

There are few comprehensive studies of the impacts of landscape-level agricultural intensification and homogenization (which tends to be accompanied by the loss of smallholder farmers) in Latin America. One review looked at studies conducted in the Argentinian Pampas, where in the late 1980s mixed cattle grazing-cropping systems were replaced by continuous cropping of a few crops. This corresponded to increased use of no-till technology (mostly with genetically modified cultivars) and an increase in field size, decreasing landscape heterogeneity and led to dramatic reductions in biodiversity in the region. Direct evidence of negative effects was found for rodents and crop-associated insects, especially non-herbivorous insects. The authors of the review by Medan *et al.* suspected that there had been net negative effects for avifauna, but the results to date were mixed ^128^. It was found that the loss of ecological heterogeneity at the landscape level directly affected diversity, abundance and distribution of small mammals, particularly rare species, habitat specialists and those species that needed grassland remnants for nesting and digging shelters. Increased use of pesticides had an indirect negative effect on rodents by reducing the food availability of invertebrate prey, vegetation cover and seeds. However, not all organisms were negatively affected by intensification. The review found higher abundance and richness of pollinators and suggested that native pollinators may have benefitted from resource-rich crops like sunflower and canola ^128^ *(and references therein)*.

In summary, inherent trade-offs between biodiversity conservation and farm productivity cannot be assumed ^53, 133^. A growing body of evidence indicates that landscapes dominated by small-scale and diverse farms (known as “land-sharing” or “wildlife-friendly” models ^12, 134^) may more effectively conserve biodiversity than landscapes dominated by large, energy- and input- intensive monocultures ^19, 46, 54, 55, 85, 103, 135– 137^.

### The matrix dynamic argument

Up to this point, the evidence that we have presented regarding how small-scale agroecological farms contribute to biodiversity conservation has taken a static approach to biodiversity. Most of the studies measured biodiversity in different types of farm or landscapes and compared them, implicitly assuming that what is there now was there before, and will be there in the future. This static approach would lead us to conclude that a particular system is good for biodiversity simply because a high number of species are recorded in that system, or vice versa. However, some species that are recorded in a particular habitat could be on their way to extinction (i.e., extinction debt; ^138^), and others that are not recorded could eventually get there through migration (i.e. immigration credit; ^139^). Given this, in addition to sampling biodiversity in various types of management systems and landscapes, we need to consider landscape-level dynamics because biodiversity is ultimately determined by dynamic processes such as extinction and immigration ^54^.

Local extinction is a natural process that occurs even in continuous habitats, therefore we can assume that it is prevalent, even more so, in fragmented habitats ^140– 146^. In fragmented habitats, we ^5, 54^ and others (e.g., Mendenhall *et al.* (2011) ^123^, (2012) ^147^) have argued that the biodiversity that can persist in the long term is largely determined by the quality of the matrix. The underlying ecology is grounded in the fact that a good matrix can not only provide habitat for many organisms and sustain high levels of biodiversity within the matrix itself, but also because a good matrix is one that allows movement of organisms among patches of forest and other natural ecosystems ^5, 148^. In a recent quantitative review paper Prevedello and Vieira ^149^ concluded that matrix type is important for biodiversity conservation, but that patch size and isolation are the major determinants for species diversity, persistence, population dynamics, and interactions in fragmented landscapes. However, in 91% of the studies that reported isolation as the main effect, incorporating matrix type significantly improved the explanatory power of the models, suggesting that matrix quality can reduce the patch isolation effect. They also concluded that matrix quality increases with increasing structural similarity with habitat patches. In most cases of fragmentation, the matrix is an agricultural matrix. Simulation models suggest that improving the quality of the matrix can offset extinction risk caused by losses of patch habitat of up to 60% ^150^.

In line with this, it has been suggested that agroforestry systems, such as shaded coffee and cacao, represent a high quality matrix that can facilitate inter-fragment migration among patches of forests in the tropics ^151– 153^. A similar argument has been made for Europe’s agri-environmental schemes when considering landscape level improvement ^154^. Unfortunately, few studies have empirically examined the actual movement of organisms in fragmented habitats through various kinds of agricultural matrices. In a study of the impacts of agri-environmental schemes in Europe, Delattre and colleagues ^155^ demonstrated that leaving grassy field margins, one of the features covered by the agri-environmental schemes of the Common Agricultural Policy framework of the European Union, improved inter-fragment migration of the meadow brown butterfly. For a tropical landscape, using mark-recapture techniques, Marin and colleagues ^156^ demonstrated that combined elements from traditional management, such as *Acacia* woodlots and live fences, have allowed the conservation of a rich butterfly biodiversity in forest fragments embedded in pasture in southern Mexico. A more direct estimate of inter-fragment communication is the genetic relationships of a particular species among various fragments. As far as we know, there are only two studies that have done this for a tropical agricultural landscape. Jha and Dick ^157, 158^ used genetic markers and conducted spatial analysis of pollen dispersal across a coffee matrix. Their results demonstrated the importance of a shade coffee matrix for the genetic diversity of the understory tree *Miconia affinis*.

Taken together, these studies provide strong evidence that diverse agroecological systems and mosaic landscapes of small-scale farms conserve biodiversity both at the local and landscape levels. In turn, other studies have found that biodiversity provides ecosystem services that contribute to agricultural productivity, sustainability and rural livelihoods (e.g. Hooper *et al.* (2005) ^84^ and Diaz *et al.* (2010) ^159^). This evidence, in combination with evidence of the failure of the neoliberal export-led model of agricultural development to reduce rural poverty and conserve biodiversity in Latin America, suggests that a new integrative approach is needed to simultaneously conserve biodiversity and eliminate poverty.

## Integrated biodiversity conservation and poverty reduction: the food sovereignty framework

Agroecological intensification ^160^ has been shown to produce food and maintain ecological services more efficiently than conventional monocropping systems ^161^. Critiques of the land-sharing approach suggest that smallholder, agroecological and organic farmers are unable to produce enough food to satisfy the growing global demand for food and agro-fuels. However, it may be argued that given the appropriate enabling conditions, including secure access to strategic resources for small landholdings and agricultural supports commensurate with national agricultural systems that support large-scale-industrial-agriculture, small-scale-diverse-agroecological farms can substantially contribute to present and future food needs ^46, 135, 162– 166^. In a review of 91 studies of organic agricultural systems across a range of geographic contexts, Badgley *et al.* ^165^ present evidence that organic agricultural production methods—while requiring higher labour inputs—can produce enough food to meet current food needs without expanding the agricultural land base, and that the use of a range of alternative agricultural practices could increase global food production by as much as 50%. Though controversial, this number is consistent with moving towards agroecological best practices and taking advantage of areas favourable to organic agriculture ^167^, supported by recent research in Africa ^162, 164, 166^. Finally, a recent review of the literature on agroecology and the right to food ^163^ suggests that small-scale farmers can double food production within a decade in critical regions by using agroecological production methods, and research consistently indicates that agrobiodiversity based on indigenous farmer knowledge contributes to food security ^168, 169^.

### Beyond the land-sharing/land-sparing controversy: food sovereignty and the agroecological matrix

Food sovereignty was broadly defined at the World Food Summit in 1996 as the right of local people to control their own regional and national food systems, including markets, natural resources, food cultures and production modes ^170– 172^. The framework stands in stark contrast to the agro-export based concept of food security, and argues that negative externalities, including the social welfare costs incurred by rural displacement and the loss of ecological services caused by monocropping are not calculated against the perceived high yields of agricultural industrialization ( Table 1). It postulates that small-scale sustainable farming, based on a dense agroecological matrix where communities have greater levels of security and control over the land, resources, and management regimes, has the potential to “feed the world and cool the planet” ^173, 174^. The framework elaborates, specifically for food production systems, the conceptual model of linked social and ecological systems ^5, 175^. It promotes agroecological production practices that seek to integrate traditional and localized knowledge with modern agricultural and ecological science to increase food production, support rural livelihoods, preserve genetic and cultural diversity, and conserve soil fertility and biodiversity ^159, 176, 177^. Of concern here are possible corrections to the degrading feedback loops between biodiversity loss and rural poverty traps associated with agricultural industrialization.

**Table 1.** Conventional Agriculture vs Food Sovereignty Model (adapted from Reardon and Pérez (2010) ^227^ and Rosset (2003) ^228^).

Issue	Conventional agriculture	Food sovereignty model
**Food and markets**	A commodity of trade, sold in national and international markets	A human right, secured through localized production and distribution
**Farming technology**	Industrial, petroleum-based, monocultures, input-intensive, chemical-dependent	Agroecological, low-input, diverse, specific to agroecosystem characteristics
**Knowledge base and** **dissemination**	Scientific and based on information provided by the input producers. Knowledge disseminated through extension services	A combination of scientific and local/traditional knowledge disseminated through farmer-to- farmer methodology
**Yield**	High yields based on hybrid and transgenic seeds, and high external inputs	High yields based on locally adapted varieties and agroecological methods of production
**Farmers and farm size**	Commercial farmers with large and medium size farms that respond to market forces	Smallholder and medium scale family farmers, supported by urban allies, help secure the food sovereignty of communities, regions, nations
**Agro-biodiversity**	Specialization on a few (often one) crop grown in monocultures	Diverse multifunctional systems
**Wild biodiversity**	Supports very low levels of wild biodiversity. Wildlife discouraged from field due to food safety concerns	Supports high levels of wild biodiversity
**Landscape**	Homogeneous. Tend to be dominated by large-scale farms producing a few crops. Low matrix quality that represent a barrier for inter-fragment migration of wildlife	Heterogeneous. Landscape mosaic. High quality matrix that promotes inter-fragment migration of wildlife
**Other natural resources (land,** **water, seeds)**	Extractivist. Burden of restoration often placed on society at large	Controlled locally to sustain environmental services provided, guided by inter- and intra- generational considerations
**Seeds**	A commodity of trade, patentable	Patrimony of all humanity, developed over centuries by rural communities and local experimentation
**Subsidies**	Tied to production, tends to favor large scale industrial farms	Directed to smallholder farmers to support farm diversification and agroecological practices

## Promising food sovereignty-based approaches

The food sovereignty framework has emerged in national constitutions (Ecuador, Bolivia, Nepal, Mali) and in national policies (Brazil, Cuba), building on civil-society and government led initiatives around the right to food, land redistribution, regional food procurement, and promotion of agroecological production methods ^178^. In the examples that follow, we review promising systems that demonstrate mechanisms and practices oriented towards food sovereignty that combine biodiversity conservation, food production and poverty alleviation. These examples present several important facets of food sovereignty, including a peasant-friendly institutional and economic context, secure land tenure for smallholders, interactions between livelihoods and agrobiodiversity, and the use of local and traditional agroecological knowledge and plants. We conclude with a call for focused research based on multi-disciplinary methodologies that uses a social-ecological systems approach to more effectively evaluate the synergies and trade-offs between poverty alleviation, sustainable food production, and ecological management strategies.

### Ecological land reform in Brazil

In the last two decades, Brazil’s explosive agricultural growth has exemplified the global tensions between biodiversity conservation, poverty reduction, and food production ^136, 179, 180^. The expansion of large-scale commercial agriculture—particularly the soy, beef and sugarcane sectors—has been associated with increased social inequality and environmental degradation ^181– 184^. In response, based on Brazil’s constitutional provisions for land reform, food sovereignty proponents advocate an “ecological land reform” that supports production for local and national consumption, and incorporates social and environmental goals into community settlement planning ^185^.

Between 1942 and 2004, Brazil’s agrarian reform program settled almost 800,000 families on smallholder plots across Brazil. While almost two-thirds of these settlements were located in the Amazon region, Pacheco ^186^ estimates that only 13% of Amazonian deforestation up to 2003 was attributable to smallholders in agrarian reform settlements. Since 1985, a growing percentage of settlements have been located in previously settled and deforested areas near urban centers ^177, 187^. Settling smallholders on abandoned land on plots averaging 25–50 hectares has resulted in the development of complex land use mosaics ^186^, producing a wide variety of subsistence and market oriented food and fuel crops, as well as ecological restoration activities required under the regulations for protected and reserve areas in agricultural reform settlements. This model has been shown to result in smallholder settlements that tend to be more intensive, include tree crops, and practice rotational cultivation followed by secondary forest fallows ^188– 190^. As part of a program to integrate conservation goals with rural poverty reduction, over 10% of the redistributed area was formally designated as forested environmental reserves, while an additional 13% is voluntarily maintained under forest cover by plot recipients ^191^. These areas provide important pockets for biodiversity conservation within agricultural landscapes, while also serving as a source for non-timber forest products. In addition, many settlements have undertaken ecosystem rehabilitation and reforestation activities, covering over 871,000 hectares by 2001 ^192^. For example, several agrarian reform settlements bordering protected areas in the threatened Brazilian Atlantic Forest ecosystem have been partners in the strategic protection and reforestation of forest fragments that act as wildlife corridors, facilitating seed dispersal and providing a buffer zone to protected areas ^179, 193, 194^.

Large-scale studies of Brazilian agrarian reform suggest that locating smallholder settlements near urban centers rather than in isolated frontier regions can facilitate not only improved environmental performance, but also farmer incomes and standards of living that are higher than the regional average ^75, 195– 197^. In an attempt to examine the potential trade-offs between food production, poverty alleviation and environmental degradation, Sparovek *et al.* ^195, 198^ conducted a comprehensive study of 4,340 settlements, comprised of 458,000 families, which were created through government-sponsored land redistribution between 1985 and 2001. These land reform settlements demonstrated significant regional variation in environmental quality (measured as a weighted composite of legal reserve preservation, deforestation, soil degradation, and ecological restoration), with the highest indices of degradation found in the northern Amazonian states and the lowest in traditionally settled areas of the south and center-west ^192^.

### Agroforestry and coffee farmer livelihoods in Central America and Mexico

Coffee and cocoa agroforestry systems also generate ecological, economic, and social benefits through farmers’ management of high levels of agrobiodiversity—key elements of the food sovereignty framework. Correspondingly, when coffee and cacao are produced as perennial monocrops with little or no shade tree canopy, substantially lower levels of agrobiodiversity are observed ^199^. In Central America and Mexico, research on the relationship between livelihoods provision, poverty reduction, and biodiversity conservation has been conducted in resource-poor, small-farmer coffee communities of Matagalpa, northern Nicaragua, Tacuba, Western El Salvador and in Chiapas and Oaxaca, southern Mexico ^200– 202^. Study sites in Central America contained a protected forest surrounded by an agroecological matrix dominated by shade coffee with smaller areas of annual crops. Farmers participating in these long-term studies grow coffee as their primary cash crop, along with a variety of food crops for consumption. A recent synthesis of this work shifted focus from biodiversity in coffee plantations themselves to the associated and planned agrobiodiversity that smallholder coffee households manage in the broader landscape ^202^. This approach uses the household as the first unit of analysis and then considers the broader range of plant biodiversity managed and used by each household in coffee plantations as well as food crop plots and home gardens. The livelihoods framework ^203^ was then used to analyze the contributions of plant biodiversity to coffee farm households. Livelihoods are defined as people’s capacities and means of living (e.g. food, income and assets, such as land, education etc.).

Small, individually managed farms contained significantly higher levels of shade tree diversity than larger plantations in both countries ^202^ and contained a significantly higher number and diversity of fruit and firewood trees ^200, 201^. In related studies on shade coffee-based agroforestry systems in Chiapas ^204– 206^, no apparent relationship was found between farmer income levels and shade tree abundance or species composition—belying in this case a perceived trade-off between income and biodiversity. Rather, all of the studied farmers managed their plantations to produce a diversity of shade tree products for consumption. That is, a focus on diversified, small-scale agroecological production—tenets of food sovereignty—helped provide both livelihood benefits and benefits to biodiversity.

Mexican and Central American smallholder coffee production systems show strong interdependencies connecting rural livelihoods with high levels of agrobiodiversity. Although these livelihoods remain difficult—seasonal hunger is common and monetary incomes are low—agrobiodiversity and dynamic local organizations connected to alternative trade networks have shown themselves to be important factors in buffering vulnerability to external shocks, including hurricanes and crashing coffee prices ^200, 207– 209^ (similar results were found in Nicaragua: ^210^). Diversity and multiple land use practiced by small farmers guarantee some level of food security through direct production of food products even when commercial production is not profitable. However, despite the benefits offered by such systems, especially as compared to specialized, input-intensive monocultural alternatives, they ultimately cannot be maintained, or their contributions to poverty alleviation improved, unless they are supported by subsidies, investment, higher and stable prices, and reinforcement of local capacities in order to scale up towards local and regional markets ^5, 19, 46, 209^.

### *Milpa* and wild varieties in Guatemala and Mexico

Guatemalan and Mexican peasants continue to practice a polyculture system known as *milpa* (corn intercropped with beans, squash, chillies, and many other edible and useful plants) as they have done for thousands of years. Diversified livelihoods—including the production of a variety of products from diversified agroecosystems for sale and self-consumption—helps them to guarantee food and economic security and stability and preserve non-economic cultural values ^19, 211, 212^. By preserving their traditional agricultural practices, small-scale farmers conserve not only crop resources, but also many wild varieties associated with their traditional systems, an approach to food sovereignty that emphasizes local values, autonomy, and biodiversity. In the semi-arid Tehuacan-Cuicatlan biosphere reserve in Mexico, researchers found 1,335 wild vascular plant species with one or more uses (e.g., fodder, medicinal, food, ornamental, soil control) ^213^. These species represent over half of the total regional species diversity of vascular plants, and 82% of familial diversity. Blanckaert *et al.* ^214^ found almost 150 useful weed species in the same region, with fodder weeds, for instance, cutting costs for industrial animal feed purchases and increasing survival of farm animals in times of drought. Similarly, herbs collected from maize fields in Mexico’s Toluca Valley serve nutritional, medicinal and aesthetic purposes, and their use as fodder boosts the economic returns on maize farming by 55% ^215^. In Chiapas, Mexico, Tzeltal Mayans can recognize more than 1200 species of plants, many of which contribute to their livelihoods ^216^. The use of synthetic herbicides puts this diversity at risk and affects food security; in response, farmers may leave parts of their fields unsprayed to permit continued collection of useful “weeds” ^215^. Thus traditional systems using wild varieties constitute another way that food sovereignty both encourages and depends on broad biological diversity, an approach distinct from and at times even in opposition to that encouraged in Latin America for the past 50 years ^19^.

In Mexico, researchers have examined the reasons for the persistence of cultivation of traditional maize varieties within the *milpa* by indigenous communities for domestic consumption, despite both the influx of cheaper imported corn from the U.S. under the North American Free Trade Agreement and the availability of less expensive domestic corn. Surveys of Zapotec indigenous households in the state of Oaxaca—an important center of corn genetic diversity—found that despite mean total production costs of more than 400% above the market cost of corn, families continued to plant and consume many traditional varieties instead of (or in addition to) purchasing corn, for reasons that include perceived higher quality, nutritional superiority, and cultural factors ^209, 217^. Thus, despite the threats posed by trade liberalization, the persistence of these traditional varieties helps to sustain food sovereignty, local food security, and biodiversity.

## Conclusion

In Latin America, the claim that there is an all-inclusive trap where economic poverty leads to biodiversity loss is not supported in the cases reviewed here, particularly in view of the higher biodiversity of typical smallholdings relative to large scale monoculture agriculture. Thus, efforts to help the smallholder agriculture sector escape poverty traps while stemming the tide of biodiversity loss, at least in Latin America, will require a strategy acknowledging the historical and continuing exogenous drivers of both problems. In this paper we have argued that these factors include the income and land structural biases and inequalities pervasive in the region, neoliberal policies that focus on the agro-export model and the conventional agricultural intensification that puts smallholders in a competitive but disadvantageous economic environment (paralleling and reinforcing Maru *et al.*’s 2012 synthesis of poverty traps among indigenous groups; ^2^). Food sovereignty is an approach originating from the rural poor of Latin America (and beyond) that unites efforts to address unbalanced international trade policies, historical legacies and continuation of inequality, and the continuing consolidation of agricultural modernization policies often associated with negative impacts for small-scale farmers and sustainable ecosystems. Latin American smallholders have maintained and adopted diverse strategies, mixing modern and traditional agricultural varieties and supporting significant levels of on-farm biodiversity. The high on-farm biodiversity associated with smallholder agroecological practices has been empirically tied to greater stability in income and recovery from environmental disaster (i.e., resilience) ^210, 218, 219^, greater food security ^19^, and generally positive effects for associated biodiversity ^54, 135^. While the predominant trend has turned to staples produced by industrial agriculture to boost per capita energy consumption, this strategy threatens biodiversity, the livelihoods of small scale farmers and diet quality ^53, 220, 221^. It also promotes chronic diseases, including diabetes, heart disease and obesity ^80^.

However, evidence elucidating the connections between food sovereignty and its emphasis on diverse traditional crops, wild plants and animal species maintained by small-scale farmers with broader economic and health benefits is still accumulating. Although many traditional systems in Latin America have proved their durability in the long term ^19^, researchers face serious methodological challenges inherent in measuring the relationship between biodiversity and food security within a common framework ^222– 224^. In emphasizing the collective right of food producers and consumers to decide the characteristics of their food system at local, regional and national levels, food sovereignty contains a crucial ambiguity—that is, the question of how to resolve possible contradictions within these different geographies, from the nation-state to the individual ^225^. This ambiguity arguably reflects both the empirical reality of immense variation between different sustainable and egalitarian institutions, and the conceptual flexibility necessary to create them.

For example, Ostrom’s decades of work (e.g., Ostrom and Nagendra (2002) ^65^ and Ostrom (2009) ^226^) have shown that local institutions are crucial for the management of the commons. Her work has also consistently emphasized that devolving power to local stakeholders is never a panacea, nor is there a guaranteed formula. However, there are certain patterns that characterize successful local institutions, an empirical observation shared by other researchers who have posited “deep democracy” and strong local control as necessary but not sufficient conditions for sustainability ^72^. We argue that the food sovereignty framework offers a novel methodological opportunity to align the issues of poverty and conservation within a general socio-ecological model. The cases presented here and in the growing literature on food sovereignty correspond to a growing empirical recognition of the significant power of diversified smallholder agricultural systems ^19, 46^, with all the tensions regarding institutions at multiple scales that this implies ^65^. But perhaps most crucially, the food sovereignty framework represents an opportunity for those concerned with biodiversity conservation and poverty to work in alliance with millions of small-scale farmers and their supporters.
