# Supplementary material for: Food sovereignty: an alternative paradigm for poverty reduction and biodiversity conservation in Latin America
Source: F1000Res. 2013 Nov 6;2:235. [Version 1] doi: 10.12688/f1000research.2-235.v1 (PMC3869480; doi:10.12688/f1000research.2-235.v1)
Supplement: Peer review history

# Peer review: Referee response for version 1

**Published:** 2013-12-10

**Authors:** Ryan Isakson

**Affiliations:** 1 Centre for Critical Development Studies, University of Toronto Scarborough, Toronto, Canada

Drawing upon a systematic review of existing literature, this article evaluates the popular claim that the food sovereignty agenda will not only democratize food provisioning, but also alleviate the poverty of food producers and encourage the conservation of biodiversity in agrarian landscapes. In so doing, it represents a valuable contribution to the academic debate and carries important ramifications for envisioning and implementing future agricultures. The authors effectively deploy the concept of poverty traps to challenge the common claim that environmental degradation, including biodiversity loss, is an inevitable consequence of rural poverty alleviation, thereby contributing to a growing literature demonstrating that economic and political democracy can, in fact, promote sustainable agricultural practices (see, for example James Boyce’s work on natural assets). Their survey of the relevant literature also demonstrates how two tenants of the food sovereignty framework, small-scale peasant-based agriculture and agro-ecological practices, are associated with higher levels of biodiversity and more resilient food systems. Overall, it’s an insightful article that effectively and concisely analyzes the relevant literatures in the social and natural sciences. 

 

The title and abstract of the article are appropriate and accurately reflect the content of the paper. The article is logically structured and well-written. The analysis is impressive and draws upon a comprehensive review of the relevant literatures.  It’s an important contribution and I highly recommend its indexing.

I have read this submission. I believe that I have an appropriate level of expertise to confirm that it is of an acceptable scientific standard.

# Peer review: Referee response for version 1

**Published:** 2013-11-08

**Authors:** Frederick Kirschenmann

**Affiliations:** 1 Leopold Center for Sustainable Agriculture, Iowa State University, Ames, IA, USA

This is one of the most succinct, yet comprehensive analysis of the complex issues surrounding the development of a resilient, socially viable food system for our future I have ever come across. Consistent with the observations made in recent UN studies, “ *Agriculture at a Crossroads*,” “ *Agroecology and the Right to Food*,”  “ *Save and Grow*” and “ *The Future We Want*” this brief paper outlines the key issues that must be incorporated into designs for a viable food system for the future. 

 

The tile and abstract accurately reflect the content of the paper and its core position. The paper clearly points out that people in their own communities, and especially small-holder farmers, need to have access to fundamental resources to have the right to food and the necessary information and natural and social capital to achieve food sovereignty, escape the poverty traps that capture so many of the very people who can provide secure food systems and restore and maintain the biodiversity necessary for a resilient food system for future generations. This paper presents a brilliant, science-based alternative paradigm to the neo-liberal, global-export-oriented model which is often presented in our current culture as “the only way to feed 9 billion people” when in point of fact, it fails to address some of the key problems of that system: entrenching the poor in  poverty traps, eroding the ecological capital of the very communities it purports to feed, and perpetuating many of the social dysfunctions that prevent large populations in poor rural communities from achieving the right to food.

 

This paper and many of those it cites need to be published widely and called to the attention of the public press so that the general public can become more aware of the issues we all need to address. This paper conforms to our highest scientific standards and makes its case persuasively and competently. I highly recommend it for indexing as presented.

I have read this submission. I believe that I have an appropriate level of expertise to confirm that it is of an acceptable scientific standard.
